# Guest-editing under the spotlight

**DOI:** 10.1098/rstb.2023.0478

**Published:** 2024-02-26

**Authors:** Richard A. Dixon, John Dainton

**Affiliations:** ^1^ BioDiscovery Institute and Department of Biological Sciences, University of North Texas, Denton, TX 76203, USA; ^2^ STFC Daresbury Laboratory, Cockcroft Institute, SciTech Daresbury, Warrington WA4 4AD, UK; ^3^ Department of Physics, University of Lancaster, Lancaster LA1 4YB, UK

Earlier in 2023, the Committee on Publication Ethics (COPE) published a discussion document entitled ‘Best Practices for Editing Collections' [1]. The document was written in response to concerns that have been raised about the quality of content in themed collections, highlighted by the fact that large numbers of articles published in such issues have had to be retracted [2]. Themed collections are becoming more prevalent in the publishing industry for a number of reasons; for example, aggressive commissioning tactics by some publishers; the corruptive effect of ‘publish or perish' for researchers; and simply because increased website functionality allows publishers to collate otherwise unconnected research articles that nevertheless cover a similar topic, into a ‘special collection'. Because guest-edited collections form the basis of publications in both *Phil. Trans. A* (physical sciences) and *Phil. Trans. B* (biological sciences), we consider that now is an opportune moment to reflect on these journals' publishing model in the context of ongoing discussions on publishing ethics and strategies.

Since the 1990s, *Phil. Trans. A* and *B* have used the guest-edited issue model. Some of the issues record key presentations at scientific meetings, most of which have been arranged by the Royal Society following prior review by the Society's Hooke Committee. Other issues are solicited from field leaders and high-quality scientists by members of the Editorial Boards, the Commissioning Editors or the Editors-in-Chief, and a number also result from unsolicited approaches to the journals. All themed issue proposals that are submitted to the journals via these avenues undergo extensive peer review by external reviewers and members of the journals' Editorial Boards. The discussion in the COPE document was primarily targeted towards journals that publish a mixture of article collections and single articles, with the warning that publishing a large number of guest-edited article collections can lead to concern about the independence, impartiality and credibility of the journal in the absence of strict and transparent oversight by the Editors-in-Chief, senior editorial board members and in-house publishing team. Although it is never possible to guarantee that scientific misconduct will not happen, we consider that *Phil. Trans. A* and *B*, with their long history of specifically publishing themed issues, already have the recommended safeguards in place and that they serve as models for how to handle the solicitation, review and publishing of article collections. More information about this can be found in a recent blog post https://royalsociety.org/blog/2023/04/theme-issue-publishing-royal-society/.

If guest-edited collections are managed with an eye to aiding scientific discourse rather than simply filling journal pages, and are rigorously reviewed and edited, we see several advantages in their favour. They can provide cutting-edge yet balanced views of a particular field that enhance the development of ideas and understanding within the field. Equally important, they can enable early career scientists who guest edit for the journals to gain exposure as conceptual leaders in their fields. Early career scientists are often thrown in at the deep end, with little experience or support in handling editorial duties/problems. The special help that the *Phil. Trans.* editorial team provides to young scientists learning the art of guest-editing ‘on the job' is a valuable feature of the journals.

Over the past decade, both primary research papers and review articles have become more susceptible to peer review manipulation, financial conflicts of interest, nepotism and nefarious actors such as paper mills and citation cartels. Rapid developments in Artificial Intelligence (AI), such as ChatGPT, have added to the concerns. No journal has all the answers to these problems, so careful monitoring at all stages from inception to publication is very important. To assist guest editors in obtaining quality reviews of individual articles, *Phil. Trans. B* will soon be implementing Transparent Peer Review, with reviews and the authors' responses being published at the time of publication of the article. The journals have also developed policies that allow the limited use of AI, but only where appropriate and transparently acknowledged by the authors. For the reality is that AI has become an increasingly important and versatile tool in the physical and biological sciences. Its history can be traced back over decades to the development and application of cutting-edge computational technologies in data-intensive experimental and theoretical physical and engineering science. Nowadays, AI versatility extends to providing textual content, which relies on working from existing material and is therefore open to substantial abuse, not least in respect to aspects of plagiarism. Journal policies must adapt to ensure that the peer review process is able to identify inappropriate use of these tools.

The *Philosophical Transactions* have a long history (since 1660) of providing a forum for scientific debate. Nowadays the need is greater than ever before for a venue that publishes high-quality collections which provide a synthesis of ideas in an increasingly complex, often interdisciplinary, scientific landscape. The problems with publication of themed issues have in many ways arisen because of the largely for-profit nature of the publishing business. The Royal Society is an independent, non-profit organization (https://royalsociety.org/journals/publishing-activities/publishing-values/), enabling its focus to remain on scientific quality rather than quantity. Many fields within the physical and biological sciences view *Phil. Trans.* as the favoured venue for publishing high impact volumes that report major advances and set new directions in the conceptual development of their fields. This esteem is at the root of the high reputation, and thereby continuing success, of the A and B issues of the journal and, as its distinguished history has already demonstrated, this reputation for quality will continue to underpin its future as new challenges of digital information technology in scientific publication, most notably involving AI, inevitably emerge. It is our hope that the above explanation and reaffirmation of our aspirations and procedures will sustain and enhance further the confidence of the global scientific community to continue to take advantage of the unique publication opportunities for their disciplines that *Phil. Trans.* facilitates, benefiting from the compliance of the editorial policies of the journals with the recommendations of the COPE discussion document.

## Data Availability

This article has no additional data.
